# The efficacy and safety of intraoperative acute normovolaemic haemodilution in complex spine surgery in a private surgical facility in Ghana

**DOI:** 10.4314/gmj.v55i1.2

**Published:** 2021-03

**Authors:** Irene Wulff, Henry O Duah, Audrey Oteng-Yeboah, Henry O Tutu, Kwadwo P Yankey, Aba S Essel, Harry Akoto, Oheneba Boachie-Adjei, FOCOS Spine Research Group

**Affiliations:** 1 FOCOS Orthopaedic Hospital, No. 8 Teshie Street, Pantang, Accra. P. O. Box KD 779, Accra-Ghana; 2 Korle-Bu Teaching Hospital, P. O. Box KB 77, Accra-Ghana

**Keywords:** Acute Normovolaemic Haemodilution, blood conservation, allogeneic transfusion, Severe Spine Deformity Surgery, transfusion reaction

## Abstract

**Objectives:**

To assess the safety and clinical benefits of intraoperative acute normovolaemic haemodilution (ANH) in complex spine surgery.

**Design:**

Prospective comparative cohort study

**Setting:**

A private orthopaedic hospital in Ghana

**Patients:**

Seventy-six patients who underwent complex spine deformity surgery

**Interventions:**

Patients were randomly assigned to two groups. 45 patients to the acute normovolaemic haemodilution (ANH) or Group 1 and 31patients to the non-ANH or Group 2. Following anesthetic administration and before incision, autologous blood was collected from patients in Group1 and was reinfused during/shortly after surgery while patients in Group2 were transfused with compatible allogeneic blood intraoperatively.

**Main Outcome Measures:**

Changes in haemodynamic parameters and incidence of allogeneic transfusions and related complications.

**Results:**

The mean age (years), gender ratio, deformity size and aetiology, fusion levels, and operative times were similar in both groups. Blood loss (ml) of patients in groups 1 and 2 were 1583ml± 830.48 vs 1623ml ± 681.34, p=0.82, respectively. The rate of allogeneic blood transfusion in groups 1 and 2 were 71% vs 80.65%, p=0.88, respectively. Haemoglobin levels (g/dL) in groups 1 and 2 were comparable in both groups at Post-operative Day (POD) 0 and POD 1. Incidence of minor allogeneic transfusion reaction was 1/45 vs 1/31, p=0.80, group-1 and group-2, respectively.

**Conclusion:**

Acute normovolaemic haemodilution can be safely performed in complex spine surgery in underserved regions. However, its use does not obviate allogeneic transfusion in patients with complex spine deformities in whom large volumes of blood loss is expected.

**Funding:**

None declared

## Introduction

The magnitude and complexity of spine procedures being performed has been increasing worldwide and Ghana is no exception. These major spine surgeries are associated with significant blood loss requiring perioperative blood transfusion, and the volume of allogeneic blood transfused is significantly related to the aetiology and complexity of the surgical procedure and surgical time.^1^,^2^ Allogeneic blood transfusion carries the risks of transmitting infections (viral and bacterial), haemolytic transfusion reactions, transfusion-related lung injury, and increases hospital costs. 3

The need for blood and blood products in the management of hospitalized patients is usually far above the available units of blood products in the blood banks of many healthcare centres in Ghana.

Often times, priority is given to trauma patients, obstetric cases, and patients with other surgical and medical emergencies in the allocations of the limited blood and blood products available. Elective surgical spine procedures are thus often delayed, rescheduled or cancelled due to unavailability of blood and blood products.

This leads to prolonged waiting periods for such cases and potentially increases undue rate of morbidity and mortality. Adequate supply of safe blood for perioperative transfusion has been a challenge in sub-Saharan Africa.^4^,^5^

A variety of blood conservations techniques are employed in complex spine surgeries including hypotensive anesthesia, antifibrinolytics, intraoperative cell salvage and acute normovolaemic haemodilution (ANH). 6 Acute normovolaemic haemodilution is a blood conservation technique which involves the removal of blood from a patient shortly after induction of anaesthesia, with maintenance of normovolemia using crystalloid and/or colloid replacement.^7^,^8^ Although ANH can also be done pre-induction, it is totally painless when the patient is asleep after induction. The amount of blood removed varies between one and three units (450 -500 mL constitutes one unit), although larger volumes may be withdrawn safely in certain circumstances. The primary objective of this blood conservation technique is to lower the patient's haemoglobin concentration during surgery so as to minimize the effect of surgical blood loss (haemodiluted blood shed during surgery has less haemoglobin than it would have without intraoperative haemodilution). The previously withdrawn whole blood is infused into the patient during or shortly after the surgical procedure. Acute normovolaemic haemodilution is usually used as a blood conservation technique combined with intraoperative cell salvage, antifibrinolytics (in this case tranexamic acid), and hypotensive anaesthesia.

Although there is a growing literature on the use of ANH in spine surgery 2,9–11, its efficacy, safety and cost effectiveness in complex spine deformity surgeries has not been adequately studied in underserved regions such as West Africa. Given the paucity of literature on the efficacy and safety of the utilization of ANH as a blood conservation method in complex spine surgery in the developing world, the present study sought to investigate the safety and efficacy of ANH and associated transfusion related complications in complex spine surgery in a private orthopaedic hospital in Ghana.

## Methods

Following approval from the institutional review board (IRB) of the Noguchi Memorial Institute for Medical Research (NMIMR), College of Health Sciences, University of Ghana, Legon (Study protocol number 046/15-16 *amend*. 2018), this prospective study was carried out on two patient cohorts who underwent complex spine deformity surgery at the FOCOS Orthopaedic Hospital, Accra-Ghana from December, 2015 to September, 2017. A full explanation of the ANH process was given to the patient during preoperative anaesthetic assessment.

Written informed consents were obtained from all patients who were 18 years and above; parental and child assents were sought for children under 18years. Patients were randomly assigned to Group1 (ANH group) and Group2 (Non-ANH group). Inclusion criteria were patients aged ≥ 8years with a pre-op haemoglobin (Hb) levels ≥12 (g/dL), body weight ≥20kg; and patients with evidence of coagulopathy and medical conditions such as cardiac and renal diseases were excluded from the study.

Baseline parameters obtained were curve aetiology and magnitude, body mass index (BMI), pre-operative haemoglobin (Hb), haematocrit, clotting profile including prothrombin time (PT), activated partial thromboplastin time (APTT), international normalized ratio (INR), and the calculated Estimated Blood Volume.

Randomization of patients into groups was done prior to the induction of anaesthesia by balloting; a random person from the surgical team was asked to randomly select a paper from an envelope containing pre-labeled folded sheets of papers with inscription “Yes” or “No”. Fifty “Yes” and 50 “No” folded sheets were printed. A patient was assigned into Group1 (ANH Group1) if a “Yes” sheet of paper was picked and into Group2 if a “No” sheet of paper was picked. Randomization was continued for 22 months until a total of 45 patients and 31 patients were enrolled into Group1 and Group2, respectively. Using the power and sample size calculating software 12, we were able to determine that this sample size had adequate statistical power for analysis (alpha=0.05 N=45 , P_0_=0.28 , P_1_=0.79 , M=0.69, Power for uncorrected chi-squared test= .998, where “alpha” is the probability committing a type 1 error, “N” is the number of participants in the ANH groupof the current study, “M” is the ratio of number of participants in the control Groupto ANH group for the current study and P_0_ and P_1_ are the reported probability of allogeneic transfusion in an ANH group and control group in a previous study^10^, respectively).

In the operating room, intravenous access was obtained with an appropriate size cannula (from gauge 16–20) depending on the size of patient and ease of venous access. The patient was connected to a multiparameter patient monitor. This provided the baseline parameters such as non-invasive blood pressure (NiBP), electrocardiogram (ECG) and peripheral venous oxygen saturation (SpO2) which were measured and recorded. End-tidal carbon-dioxide concentration (EtCO2) were measured and recorded after intubation from the multiparameter patient monitor. The patient was pre-oxygenated for 3 minutes and general anaesthesia was induced intravenously with midazolam (0.1mg/kg), propofol (5–10mg/kg), and fentanyl (5mcg/kg).

These provided excellent intubating conditions without the use of muscle relaxant per institutional protocol. Laryngoscopy was done and the trachea was intubated with appropriate-sized endotracheal tube; adhesive tape was used to secure the tube in place after equal air entry had been ascertained. In cases where there was a drop in blood pressure, ephedrine 2.5–5mg was administered. As per institutional spine protocol, anaesthesia was maintained with total intravenous anaesthesia (TIVA) using propofol and fentanyl titrated to effect. After induction of anaesthesia and stable cardiovascular parameters, a second venous access (gauge 16–20) as well as an arterial access (gauge 20) were secured.

Before the start of surgery, autologous blood was collected under gravity from patients in Group 1 via the arterial access into standard transfusion bags containing the anticoagulant, citrate-phosphate-dextrose-adenine (CPDA) solution, under strict asepsis. A male-to-male connector was used to connect the transfusion bag to the arterial access. At the same time, the replacement fluid which was 6% hydroxyethyl starch was given through the venous access at a rate matching the rate of collection of autologous blood.

A weighing scale was used to collect the calculated volume of autologous blood. The CPDA/autologous ratio in the transfusion bag was 1: 10. The volume of autologous blood withdrawn was calculated from the Gross formula below. 13 For the purpose of this study a final and anticipated Hemoglobin of 11.0g/dL was selected to calculate the volume of blood to be removed from the patient.


$$\text{Volume=}\frac{{\text{EBV x (H}}_{\text{i}}{\text{-H}}_{\text{f}}\text{)}}{\text{Havg}}$$

EBV = Estimated blood volume of the patient (70ml/kg)

Hi = Initial haemoglobin

Hf= Final haemoglobin

Havg = average haemoglobin (Hi + Hf)/2

The transfusion bag was gently shaken frequently to adequately mix the anticoagulant with the blood, avoiding clot formation. Once the required volume of blood was collected, the collecting tubing was clamped and knotted. The arterial access was flushed with heparinized saline and connected to the pressure transducer system for monitoring of invasive blood pressure which is a prerequisite for intraoperative spinal cord monitoring. The bag was then labelled with the patient's name, date and time. If more than a unit was taken, the bags were sequentially numbered as they were filled. “AUTOLOGOUS” was also written in red on the label. The blood was kept in the operating room and kept at room temperature for up to 4 hours. Haemoglobin (Hb) level was measured after withdrawal of blood.

Once the period of major blood loss was over or earlier if indicated, the blood was transfused to the patient in the conventional manner. If more than one unit was taken, the units were transfused in reverse order of collection. Tranexamic acid (TXA) was administered to each patient prior to surgical procedure to reduce intra-operative bleeding using a loading dose of 10mg/kg and followed by infusion of 1mg/kg/hour.

Intra operative (intra-op) parameters included duration of surgery in minutes, mean level of vertebrae fused, estimated blood loss (EBL) in mL, % volume of blood loss, use of intra-op cell saver and volume transfused in mL, and units of blood products given. Post-operative (Postop) Hb, Haematocrit, and clotting profile were measured sequentially immediately after surgery (ie. Postoperative Day (POD): POD 0, POD 1 and POD 2). Number of postop units and volume of allogeneic blood transfused were noted for each patient. Other information obtained were duration and amount of wound drainage, length of stay, and transfusion events. Patients were transfused when their hemoglobin was ≤ 7g/dL.

Data was initially collected on case report forms (CRFs) but later recorded in Microsoft excel and exported to Stata 14 for analysis. The two main outcomes under investigation were the efficacy and safety of ANH. Efficacy of ANH was assessed in relation to the adequacy of ANH in replacing blood loss without the need for additional allogeneic transfusion. Thus, the specific outcome measures under investigation were changes in haematologic and haemodynamic parameters and incidence of allogeneic transfusions and related complications between the groups. By employing a comparative descriptive and inferential analysis using frequency tables, chi-square test for independence, paired t-test and independent t-test, we compared the incidence of allogeneic blood transfusion and transfusion-related complications between the two groups. We also assessed the change in the preoperative and postoperative haemoglobin concentrations, haematocrit, platelet counts and clotting profiles of patients between the two groups. Categorical variables were presented as frequencies and column percentages. Continuous variables were reported as mean ± standard deviation. Statistical significance (denoted ** in the tables) was pegged at 0.05 alpha level.

## Results

### Baseline Parameters comparison

A total of 76 patients were included in the study, 45 in Group 1 and 31 in Group 2. Gender ratio were similar, 26M/19F vs 13M/18F for Groups 1 and 2, respectively= 0.243. The age range were 8–27 years and 9–26years for Group 1 and Group 2, respectively. The average age, BMI, pre-operative coronal and sagittal cobb angles were similar in both groups. The ranges of the pre-op sagittal cobb were 11o-240o and 10o-256o for Group 1 and Group2, respectively. Spine deformities of idiopathic aetiology constituted 53.33% and 48.39% of patient in Groups 1 and 2, respectively.

The mean pre-operative haemoglobin and haematocrit were significantly higher in Group1 compared to Group2: 13.54 ± 0.99 g/dL vs 12.92 ± 0.72 g/dL, p= 0.002 and 0.401 ± 0.03 vs 0.377 ± 0.03, p<0.001, respectively. However, the pre-operative PT, APPT and INR were comparable in Group1 and Group2. The estimated blood volume for patients in Group1 and Group2 were 2922.18mls±978.73 vs 3049.29mls ±665.47, p= 0.53. Details have been summarized in Table 1.

**Table 1:** Comparison of socio-demographic and baseline clinical parameters of patients between the study groups

Characteristics	ANH Group N=45	Non-ANH N=31	p-value
**Gender: Male/Female**	26/19	13/18	0.243
**Age**	15.31 ± 4.10	15.45 ± 3.77	0.87
**Pre-op coronal cobb (^0^)**	90.00±34	85.5±34.54	0.62
**Pre-op sagittal cobb (^0^)**	90.48±51.86	88.03±54.62	0.84
**Aetiology of Deformity**			
**Idiopathic**	24 (53.33%)	15(48.39%)	
**Congenital**	6(13.3%)	6(19.35%)	
**Post Tuberculous(TB)**	7(15.56%)	5(16.13%)	
**Neurofibromatosis**	6(13.3%)	1(3.23%)	
**Neuromuscular**	2(4.44%)	3(9.68%)	
**Other**	-	1(3.23%)	0.44
**Body Mass Index (kg/m^2^)**	19.46 ± 2.99	19.64 ± 2.73	0.80
**Pre-Op Haemoglobin** **(g/dL)**	13.54 ± 0.99	12.92 ± 0.72	0.002**
**Pre Op Haematocrit**	0.401 ± 0.03	0.377 ± 0.03	<0.001**
**Pre-op PT (s)**	13.07 ± 1.11	13.09 ± 1.03	0.94
**Pre-Op Platelets (x10^9L)**	272.02 ± 52.00	276.25 ± 64.58	0.75
**Pre-Op APTT (s)**	31.39 ± 1.57	31.05 ± 1.25	0.39
**Pre-Op INR (s)**	1.08 ± 0.09	1.09 ± 0.08	0.82
**Estimated Blood Volume** **(ml)**	**2922.18±978.73**	**3049.29±665.47**	**0.53**

### Intra operative parameters

Intraoperatively, no significant differences were observed between Group 1 and Group 2 with regards to average duration of surgery, level of vertebral fusion, estimated blood loss (EBL), percentage estimated blood volume (EBV) loss and mean arterial pressure (MAP). Details have been summarized in Table 2.

**Table 2:** Comparison of duration of surgery and intra operative parameters between the study groups

Characteristics	ANH Group (N=45)	Non-ANH (N=31)	p-value
Fusion Levels	12.7 ± 2.31	12.4 ± 2.95	0.55
Duration of Surgery (min)	223 ± 70.95	238 ± 70.05	0.36
Volume of transfused cell salvaged (ml)	540 ± 302	560 ± 223	0.76
Estimated Blood Loss (ml)	1583 ± 830.48	1623 ±681.34	0.82
Percentage (%) of EBV loss	58.32±32.01	57.89±29.90	0.95
Intra operative MAP (mmHg)	**71.77±6.12**	**73.42±4.57**	**0.41**

### Comparison of postoperative blood requirements

The proportion of patients who received allogeneic blood transfusion and the volume transfused (whole blood + fresh frozen plasma (FFP) + packed cells) were 71.11% vs 80.65%, p=0.88 and 743ml ± 638.99ml vs 861.83ml ± 644.19ml, p=0.43 in groups 1 and 2, respectively. Fresh Frozen Plasma, Whole Blood and Packed cells transfusions were similar in both groups (Table 3). There was no significant difference in the Hb levels and haematocrit between the two groups on the Post-Op Day 0 (immediate post-op) and Post-op day 1 (POD 1). Likewise, the clotting profile (PT, APTT and INR) was comparable between the two groups at POD 0 and POD1. Both groups recorded one incidence of minor transfusion reaction following allogeneic blood transfusions. Details are reported in Table 3.

**Table 3:** Comparison of postoperative blood requirements and the serial clotting profiles between the study groups

Characteristics	ANH Group N=45	Non-ANH N=31	p-value
Proportion who received allogeneic blood	32(71.11%)	25(80.65%)	0.88
Volume of transfused allogeneic blood (mL)	743 ± 638.99	861.83 ±644.19	0.43
Volume of transfused Whole blood (mL)	389.4 ±232.2	491.9±215.4	0.15
Volume of transfused Fresh frozen plasma (FFP) (mL)	594.3±424.7	566.9±405	0.79
Volume of transfused Packed Cells (ml)	161.5±232.3	331.2±201	0.14
Post-Op Day Zero Haemoglobin (g/dL)	9.4 ± 1.14	9.6 ± 1.00	0.34
Post-Op Day Zero Haematocrit	0.278±0.04	0.285 ± 0.03	0.42
Post-Op Day Zero PT(s)	15.07 ± 1.66	15.45 ± 3.20	0.48
Post-Op Day Zero APTT(s)	30.48 ± 1.53	29.65 ± 3.22	0.16
Post-Op Day Zero INR (s)	1.25 ± 0.14	1.44 ± 0.10	0.70
Post-Op Day One Haemoglobin (g/dL)	10.15 ± 1.26	10.38 ± 1.30	0.45
Post-Op Day One Haematocrit	0.298 ± 0.04	0.302 ± 0.04	0.64
Post-Op Day One PT(s)	14.32 ± 1.55	14.74 ± 1.38	0.22
Post-Op Day One APTT(s)	31.00 ± 2.13	30.82 ± 1.32	0.77
Post-Op Day One INR(s)	1.19 ± 0.13	1.21 ± 0.11	0.41
Transfusion Reaction	1/45	1/31	**0.80**

## Discussion

This study showed that fewer patients in the ANH group received allogeneic blood compared with the non-ANH group and lower volume of allogeneic blood was transfused to the patients in the ANH group intraoperatively and postoperatively. Nevertheless, these observed differences did not reach statistical significance implying that ANH did not obviate allogeneic transfusion in our patient cohort with severe spine deformities. Zhou et al 14 in their meta-analysis involving 63 different studies demonstrated the efficacy of ANH in a wide range of surgical specialty surgeries although they also reported significant methodological bias inherent in the reviewed papers which raises concerns about the actual efficacy of ANH.

Contrary to our findings on the efficacy of ANH, Verma et al 9 demonstrated the efficacy of ANH in 70 patients who were diagnosed with adolescent idiopathic scoliosis (AIS) and underwent spine surgery at the Royal Manchester Children's Hospital. Their findings revealed significantly higher rate and volume of allogeneic blood transfusions in the controls group compared to the ANH group. The incongruence in findings may be due to difference in aetiology and complexity of spine deformity. Blood loss in spine surgery is dependent on the underlying spine disorder as well as the surgical complexity and is significantly higher in patients with neuromuscular disorders or patients undergoing three column osteotomy procedures compared with those managed for mild to moderate adolescent idiopathic scoliosis or patients undergoing posterior-only procedures without any 3 column osteotomies. It is also positively associated with duration of surgery, vertebral fusion levels, as well as in adult patients compared with paediatric patients undergoing similar procedures.^2^,^15^ Shapiro and Sethna 2 in their systematic review reported that the average EBL in most studies ranged 750–1,500ml among AIS patients who undergo posterior fusion whereas the average EBL range was higher in neuromuscular scoliosis (2,000–3,500ml). The authors also reported an average EBL range of 65–150 ml per vertebral levels fused in AIS with higher average EBL recorded per vertebral fusion in neuromuscular scolisis. Moreover, Ialenti et al 16 reported an average EBL of 907±775ml for posterior surgeries and 1277±821mL for combined posterior and anterior surgery in AIS patients with mean scoliotic curvature <70o. These highlight the effcet of underlying aetiology and curve complexities on the degree of blood loss. In the study by Verma et al 9 , their patients included only AIS patients. Moreover, AIS patients from the developed regions are usually diagnosed and treated early while the curves are relatively small. The utilization of ANH in such patients will likely obviate allogeneic blood transfusions as blood loss during spinal reconstruction for smaller curves is associated with reduced blood loss. However, our patient population consisted of patients with heterogeneous etiologies including idiopathic, congenital, neuromuscular, neurofibromatosis, and posttuberculous amongst others as seen in Table 1.

Moreover, spine deformity patients in underserved regions such as Ghana usually report to health facilities very late in the course of the disease when their curves are already severe. Thus, late presentation is associated with severe spine deformities and significant blood loss. Nugent et al 17 reported that Cobb angle measurements >70degree and surgical blood loss >1400ml were associated with 4 times and 3 times greater risk of blood transfusion, respectively. In the present study, the average coronal Cobb measurements were 90.00±34degree and 85.5±34.54degree for the ANH and non-ANH groups, respectively. The average sagittal Cobb also measured 90.48±51.86degree and 88.03±54.62degree in the ANH and non-ANH groups, respectively. A relatively high EBL was observed for patients in both the ANH group (1583 ± 830.48ml) and the non-ANH group (1623 ± 681.34ml) which is higher compared to other studies which reported an average EBL of less than 1100ml for posterior spinal fusion (PSF) surgery in patients diagnosed with AIS. 16,18 This coupled with the average fusion levels of 12 levels in each group commensurate the degree of estimated blood volume (EBV) loss in the present cohort (>57% EBV in both groups). Our patient cohort included cases with severe spine deformity in whom the surgical blood loss during surgery was very significant that the benefit of ANH was most likely mitigated. Sharma et al 19 recommend that acute blood loss greater than 30% EBV is an indication for blood transfusion.

de Oliveira et al 10 also investigated the efficacy of ANH among patients in idiopathic scoliosis and reported a significantly lower rate of allogeneic transfusion in an ANH group (28%) compared to 79% allogeneic blood transfusion in their control group. Their study was performed in patients with idiopathic scoliosis whereas the aetiology in the present study was heterogenous consisting of idiopathic, congenital, post TB, neuromuscular, neurofibromatosis and syndromic aetiologies. Blood loss is relatively lower for idiopathic compared to the other aetiologies 2 hence this may partly explain the variations in the efficacy of ANH.

Consistent with our findings, a previous experimental study by Hasan et al 11 showed that ANH does not provide significant additional benefits when combined with intraoperative cell salvage (ICS). The congruency in findings may be explained by similarity in aetiology, degree of surgical blood loss, and levels of spine fusion in both studies. There was no significant difference in the intraoperative MAP between the two groups in our study. We reported no cardiac event in the ANH group. No transfusion reaction was recorded during reinfusion of withdrawn autologous blood in the ANH group. We also report no significant difference in the incidence of minor transfusion reactions following allogeneic transfusions between the two groups. Our finding on safety of ANH was in agreement with previous studies which have reported that the utilization of ANH is safe in spine deformity surgery. 9–11

Our findings indicate that for severe spine deformity patients who are candidates for corrective spinal surgery but have personal, religious and cultural reservations for allogeneic blood transfusion, care should be taken as allogeneic blood transfusions may be inevitable for their complex surgeries despite the use of ANH. For these patients, we would recommend that in addition to the intraoperative blood conservation techniques their surgeries be staged to allow adequate time to replenish blood loss and Hb prior to their final surgery. In our practice, we electively stage complex deformity surgeries as most will require high grade osteotomies which are associated with significant blood loss. From the surgical planning point of view, our experience has shown that the withdrawal of autologous blood for ANH does not delay the start of surgery because it is done after positioning while the surgical team is prepping and draping the patient. It takes approximately 20 minutes when it is obtained from an arterial line.

The shortcomings of the study may include the low number of patients recruited. The target of post ANH hemoglobin could also have been dropped to 10g/dl instead of 11g/dl. This was to prevent excessive dilution of the blood at the start of surgery, considering the significant amount of blood loss that occurs during exposure of the spine in the first hour of surgery in severe deformities. Patients with less complex deformities may benefit from dropping the Hb to 10g/dl since exposure in such patients is easier and not usually associated with significant blood loss. The benefits of ANH may be seen in this later group. A subset of patients with less complex procedures and lower blood loss was not available for review due to the small number of total patients. Future studies focus their investigation of the efficacy of ANH among AIS patients with smaller curves. Another direction for future studies is to use a larger sample to investigate the threshold of spine deformity for which the benefits of ANH can be mitigated.

## Conclusion

The study shows that acute normovolaemic haemodilution (ANH) does not significantly eliminate intraoperative or postoperative transfusion of allogeneic blood in patients with complex spine deformities in whom large volumes of blood loss is expected. This implies the need for thorough planning to mitigate intra operative blood loss and the need for adequate and safe blood products for the surgical management of complex spine deformities in the sub-region.
